# Severe thrombocytopenia in a child with typhoid fever: a case report

**DOI:** 10.1186/s13256-016-1138-6

**Published:** 2016-11-30

**Authors:** Mohammed Al Reesi, Glenn Stephens, Brendan McMullan

**Affiliations:** 1 Sydney Children’s Hospital, Sydney, Australia; 2 Department of Immunology and Infectious Diseases, Sydney Children’s Hospital, High Street, Randwick, NSW 2031 Australia

**Keywords:** Typhoid fever, Thrombocytopenia, Prolonged fever, Case report

## Abstract

**Background:**

Although thrombocytopenia is common in typhoid fever, its course, response to treatment, and need for specific therapies such as platelet transfusion are not well characterized.

**Case presentation:**

We report a case of typhoid fever in a 4-year-old Asian male returned traveler, admitted with prolonged fever and found to have severe thrombocytopenia (platelets 16 × 10^9^/L). Despite appropriate antibiotic therapy, his platelet recovery was slow, but did not lead to complications and he did not require platelet transfusion.

**Conclusions:**

There is no consensus in the medical literature guiding the optimal management of severe thrombocytopenia in typhoid fever, but it may improve with conservative management, as in our case. The epidemiology and management of this condition merits further research to guide clinical practice.

## Background

Typhoid fever is a systemic infection caused by *Salmonella enterica* subsp*. enterica* serovar Typhi and occasionally *Salmonella* Paratyphi. Thrombocytopenia is relatively common in typhoid fever, with a reported incidence up to 26% in children. It has been classified as a marker of severity in typhoid fever and indicates a high risk for development of complications [1]. Despite this, its pathophysiology and management in typhoid fever is not well established. We present a case of severe thrombocytopenia in a child with typhoid fever and we discuss the published literature.

## Case presentation

A 4-year-old Asian boy presented to the emergency department at a tertiary children’s hospital in Sydney, Australia, 1 day after returning from travel to Bangladesh with a persistent fever for 10 days. He had visited Bangladesh with his parents and stayed for 8 weeks. He had vomiting, diarrhea, and fever 3 weeks after arriving in Bangladesh, for which he was given oral ciprofloxacin for 3 days. The vomiting and diarrhea resolved after 1 week, but he continued to have intermittent fevers up to 38 °C (100.4 °F) for an additional 2 weeks. During the last 10 days of his stay in Bangladesh, his fever became persistent, with peaks of 40 °C (104 °F). He had reduced oral intake and constipation, but no vomiting. His mother reported that she had developed self-limited vomiting and diarrhea for a few days after the onset of his symptoms. The family denied eating street food or drinking tap water in Bangladesh. There was no history of contact with patients with tuberculosis. The child had been born in Australia to Bangladeshi parents, and his immunizations were up to date, according to the Australian schedule. He did not receive any travel vaccines prior to travel or malaria prophylaxis. His medical history was unremarkable apart from mild asthma.

In the emergency department he appeared unwell and moderately dehydrated. He was febrile at 39.8 °C (103.6 °F), tachycardic with a heart rate of 160 beats per minute, and his respiratory rate was 32 breaths per minute. There was no icterus, pallor, or lymphadenopathy. A skin examination did not reveal any rash, petechiae, or bruising. A chest and cardiovascular examination revealed no abnormalities. His abdomen was soft, mildly tender, and distended with no organomegaly. There was no clinical ascites and his bowel sounds were present. He was alert and oriented, with a normal neurological examination. He had no bone or joint pains or swelling. Initial investigations showed anemia, leukopenia, and thrombocytopenia. His hemoglobin concentration was 102 g/L and reached a nadir of 89 g/L on day 11 of admission. He had a nadir white cell count of 4.30 × 10^9^/L (neutrophils 2.6 × 10^9^/L and lymphocytes 0.9 × 10^9^/L) on presentation, which gradually improved to 11 × 10^9^/L by day 11. His initial platelet count was 97 × 10^9^/L. His renal function was normal apart from mild hyponatremia, while his liver function tests showed hypoalbuminemia and mild transaminitis with normal bilirubin concentrations. His C-reactive protein level was elevated at 92 mg/L. Considering his clinical presentation, travel history, and the initial investigation results, the differential diagnoses included typhoid fever, malaria, and dengue fever. We ordered a blood culture, and thick and thin blood films for malaria parasites and dengue IgM, IgG, and NS1 antigen. He was then commenced on ceftriaxone intravenously and admitted to our hospital. There were no malaria parasites seen in two films and dengue serology was also negative. The following day his stool and blood cultures grew *Salmonella* Typhi. The organism was reported to be susceptible to ceftriaxone and azithromycin, with decreased susceptibility to ciprofloxacin. It was reported to be resistant to ampicillin, chloramphenicol, and trimethoprim.

**Fig. 1:**
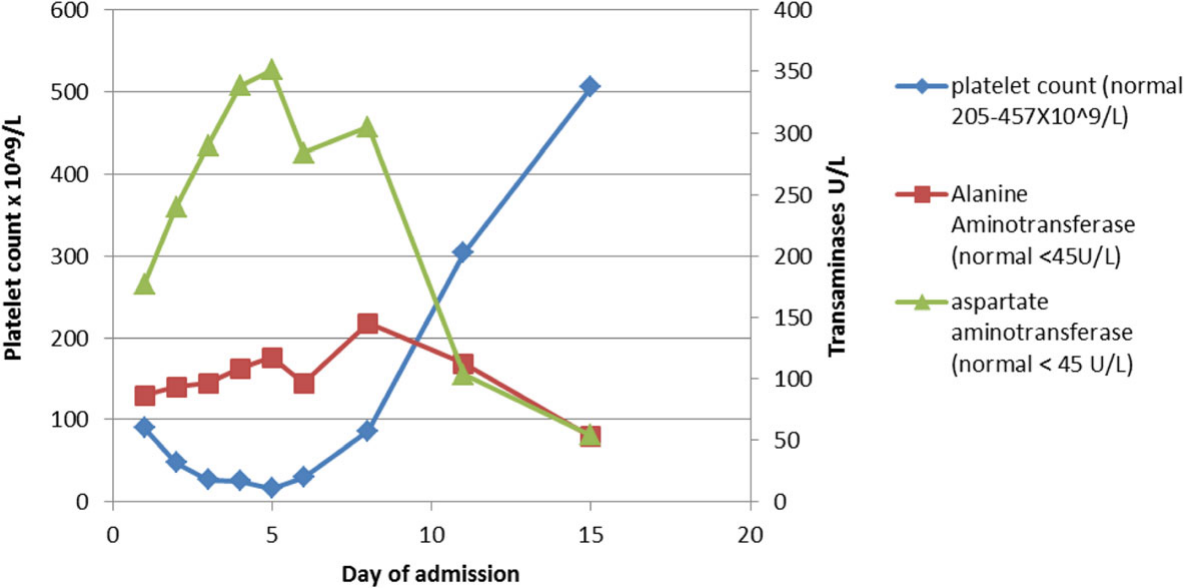
Progress of thrombocytopenia and transaminitis over time

Our patient continued to have fever spikes to 39–40 °C every 4 hours after admission until the fifth day, when the frequency of fever decreased to three spikes daily. Further improvement was noticed by day 9, with temperature spikes decreasing to twice daily and less than 39 °C. He required intravenous fluids for a short period to correct his dehydration until his oral intake gradually normalized over the first week in hospital. In addition, he received an albumin infusion on day 5 after he developed clinical ascites with a further drop in his albumin to 17 g/L. Thrombocytopenia was notable in our patient. His platelet count initially fell steadily and reached a nadir of 16 × 10^9^/L on day 5 despite appropriate antibiotic therapy (Fig. 1). He was monitored closely for complications associated with thrombocytopenia: his sensorium remained intact and he did not develop petechiae, bruising, or rectal bleeding during admission. There was no sign of intestinal perforation, with normal bowel sounds and an absence of bloody stool. At this stage, we considered whether additional therapy for thrombocytopenia would be required, such as platelet transfusion. Upon discussion with our infectious diseases team, it was decided to treat him conservatively with close observation and not to give him a transfusion. His platelet count was monitored on a daily basis, began to improve on day 6 of admission, and finally normalized on day 11 and then climbed to supra-normal levels by day 15. Likewise, his transaminases were abnormal throughout admission, peaking on day 5 (Fig. 1), but started to improve before discharge. By day 9 of admission, his oral intake improved and oral azithromycin was added to transition to oral therapy. He completed a 12-day course of ceftriaxone in hospital and was discharged in a good condition, although he still had occasional fever spikes to 39 °C. He continued to have intermittent elevated temperatures (<38 °C) at home, but his parents reported that he returned to his previous energy level and activity. After he completed a total 7-day course of azithromycin, he was reviewed in an outpatient clinic where he was afebrile with a normal examination.

## Discussion

Typhoid or “enteric” fever is a systemic infection caused by *Salmonella enterica* subsp*. enterica* serovar Typhi and occasionally *Salmonella* Paratyphi. It is characterized by severe systemic illness, often presenting with features of fever, constipation, and abdominal pain. Its incidence in endemic countries can be as high as 540 cases per 100,000 population, making it a public health challenge [1]. In an Australian setting, it particularly affects returned travelers from the Asian subcontinent [2].

Important differential diagnoses of typhoid fever in a returned traveler from South and Southeast Asia include dengue fever and malaria. In addition, acute murine typhus can have similar presentation to typhoid fever. It is common, yet underdiagnosed, in travelers from this region. Thompson *et al*. [3] found serologic evidence of acute murine typhus in 17% of patients who presented with undifferentiated febrile illnesses at a tertiary referral hospital in Nepal. All patients had sterile blood cultures with no cases of murine typhus found among those with confirmed enteric fever [3]. We did not investigate our patient for murine typhus or other rickettsial infection because typhoid fever was confirmed promptly on day 2 of his admission.

Thrombocytopenia is an important finding in the assessment of returned febrile travelers. It is defined as a platelet count below the lower limit of normal (i.e., <150 × 10^9^/L). It is further subdivided into mild (100–150 × 10^9^/L), moderate (50–99 × 10^9^/L), and severe thrombocytopenia (<50 × 10^9^/L). In a study conducted in an outpatient clinic in Germany, 3.8% of returned travelers had thrombocytopenia [4]. Typhoid/paratyphoid fever was responsible for 14% of the cases, ranking fifth after malaria, acute human immunodeficiency virus (HIV) infection, dengue fever, and Epstein-Barr virus (EBV) infectious mononucleosis. The most frequent travel destination in the study was Asia (42%) [4]. Thrombocytopenia is well reported in patients with typhoid fever. Malik [1] reported an incidence of 26% in Malaysian children with typhoid fever and Pohan [5] found it in 61.5% of cases in one study of adults. Despite this, the pathophysiology of thrombocytopenia and its clinical course in typhoid fever are not well understood. Proposed mechanisms of the hematological manifestations of typhoid fever, including thrombocytopenia, include bone marrow suppression, peripheral destruction by the reticuloendothelial system, autoimmune-induced destruction, and *Salmonella* endotoxin-induced thrombocytopenia [5, 6]. Bone marrow examination was not performed in our patient but we note he had abnormalities in three hematological cell lines, suggesting possible bone marrow suppression.

Factors that might contribute to the severity of typhoid fever include the duration of illness before therapy, the inoculum size, the immune status of the patient, and the previous vaccination against typhoid fever [7]. Our patient had been unwell for several weeks before presentation and thus the inoculum size at the time of commencing treatment could have been relatively high. He had no known or suspected immunodeficiency, but had not received a typhoid vaccination prior to travel to Bangladesh, which could have prevented this illness. The duration of illness and fever in patients with typhoid strains resistant to ampicillin, chloramphenicol, and trimethoprim can be more prolonged despite receiving antibiotics to which strains are susceptible [8]. *Salmonella* Typhi haplotype H58 predominates in many parts of Southeast Asia and is responsible for most multidrug resistance [9]. We did not perform genetic typing for our isolate, but its resistance to ampicillin, chloramphenicol, and trimethoprim, with decreased susceptibility to ciprofloxacin, suggests it may have been this haplotype.

In addition to splenomegaly and leukopenia, thrombocytopenia is considered a sign of severe disease in typhoid fever with a higher risk for development of complications. Thrombocytopenia usually develops during the course of the illness, but it can be a presenting feature of typhoid fever, as in this case. Severe complications of typhoid include intestinal perforation, intracranial hemorrhage, and multi-organ failure [6, 7]. Among 102 children with typhoid fever in one study, 33% developed complications, most commonly anicteric hepatitis and bone marrow suppression, but also paralytic ileus, myocarditis, psychosis, cholecystitis, osteomyelitis, peritonitis, and pneumonia. The rate of any complications among those with thrombocytopenia was 54% [1]. Our patient had anicteric hepatitis, hypoalbuminemia with ascites, and thrombocytopenia, but he remained alert and oriented without any clinical evidence of intracranial hemorrhage despite a platelet nadir of 16 × 10^9^/L on day 5 of admission. He also did not have any clinical evidence of intestinal perforation and his renal function remained normal throughout his admission. Considering our patient’s pancytopenia, we considered the possibility of infection-associated hemophagocytic syndrome as a complication of his typhoid fever, because this has been previously reported [10]. Given that he was slowly improving clinically, we decided to observe him closely without performing additional invasive tests, such as bone marrow examination. Further investigation, including consideration of hemophagocytic syndrome, would have been required if he had failed to improve.

Because the risk of hemorrhage is increased when the platelet count falls below 20 × 10^9^/L, this level was traditionally considered the threshold for prophylactic platelet transfusion. Later prospective studies have proved that lowering this trigger to 10 × 10^9^/L in stable patients with cancer or blood disorders is still safe. However, platelet count should not be the only indicator for deciding transfusions. Other important elements that indicate the patient is at increased risk of bleeding, and thus likely to have an increased need for platelet transfusion, include raised body temperature, sepsis, and rapid decrease in platelet count. Like most blood products, platelet transfusions are not free of adverse effects. These include blood-borne infections, although now rare owing to good screening; bacterial contamination; febrile transfusion reactions; transfusion-related acute lung injury; and anaphylactic reactions [11].

There are no studies or guidelines addressing the management of thrombocytopenia in typhoid fever. This poses a challenge for clinicians, especially when faced with severe thrombocytopenia, as in this case. Some case reports have described platelet normalization shortly after starting antibiotic therapy without a need for platelet transfusion [12, 13]. In one case, however, the platelet count fell from 154 × 10^9^/L to 14 × 10^9^/L despite antibiotic therapy, and this was associated with multi-organ failure; plasma exchange was given to correct the thrombocytopenia and other abnormalities [7]. One adult patient died from severe hemolytic uremic syndrome attributed to *Salmonella* Typhi, with failure to respond to a platelet transfusion given along with a blood transfusion and plasmapheresis [14]. Our patient had a relatively slow recovery with fever and hepatitis persisting for 2 weeks, despite appropriate antibiotic therapy, and a slow return to normalization of platelet count on day 11. However, he avoided a platelet transfusion and was well at last follow-up.

## Conclusions

We have described a case of severe thrombocytopenia in typhoid fever with slow clinical and laboratory response but complete recovery after appropriate antibiotic therapy and supportive care alone, thus avoiding the risks associated with platelet transfusions or plasmapheresis. The optimal management of thrombocytopenia in typhoid fever merits further study to guide clinical practice.
